# Core level binding energies of functionalized and defective graphene

**DOI:** 10.3762/bjnano.5.12

**Published:** 2014-02-03

**Authors:** Toma Susi, Markus Kaukonen, Paula Havu, Mathias P Ljungberg, Paola Ayala, Esko I Kauppinen

**Affiliations:** 1 Department of Applied Physics, Aalto University School of Science, PO Box 15100, FI-00076 Aalto, Finland; 2 University of Vienna, Faculty of Physics, Strudlhofgasse 4, A-1090 Vienna, Austria); 3 Present address: Helsingin matematiikkalukio, Kuusikkotie 3, 00630 Helsinki, Finland; 4 Present address: Suomen ympäristökeskus, Mechelininkatu 34a, 00260 Helsinki, Finland; 5 LOMA, Université Bordeaux 1, 351 Cours de la Libération, 33405 Talence, France

**Keywords:** core level, defects, density functional theory, graphene, X-ray photoelectron spectroscopy

## Abstract

X-ray photoelectron spectroscopy (XPS) is a widely used tool for studying the chemical composition of materials and it is a standard technique in surface science and technology. XPS is particularly useful for characterizing nanostructures such as carbon nanomaterials due to their reduced dimensionality. In order to assign the measured binding energies to specific bonding environments, reference energy values need to be known. Experimental measurements of the core level signals of the elements present in novel materials such as graphene have often been compared to values measured for molecules, or calculated for finite clusters. Here we have calculated core level binding energies for variously functionalized or defected graphene by delta Kohn–Sham total energy differences in the real-space grid-based projector-augmented wave density functional theory code (GPAW). To accurately model extended systems, we applied periodic boundary conditions in large unit cells to avoid computational artifacts. In select cases, we compared the results to all-electron calculations using an ab initio molecular simulations (FHI-aims) code. We calculated the carbon and oxygen 1s core level binding energies for oxygen and hydrogen functionalities such as graphane-like hydrogenation, and epoxide, hydroxide and carboxylic functional groups. In all cases, we considered binding energy contributions arising from carbon atoms up to the third nearest neighbor from the functional group, and plotted C 1s line shapes by using experimentally realistic broadenings. Furthermore, we simulated the simplest atomic defects, namely single and double vacancies and the Stone–Thrower–Wales defect. Finally, we studied modifications of a reactive single vacancy with O and H functionalities, and compared the calculated values to data found in the literature.

## Introduction

X-ray photoelectron spectroscopy (XPS) is commonly used to identify the relative amounts of chemical elements in a sample, and it can provide information about their chemical states, i.e., bonding. Although the method is not local, XPS is able to discern specific atomic defects if they are numerous enough and, furthermore, provide essential statistical information on their concentrations. Typically, XPS has been limited to surface characterization because of the limited escape depth of photoemitted electrons. However, for low-dimensional carbon nanomaterials such as graphene or carbon nanotubes, the escape depth exceeds the size of the system, and this makes XPS in practice a convenient bulk characterization tool.

In order to interpret the binding energies measured by XPS, a reference to which such energies can be compared is needed. Density functional theory (DFT) calculations can be employed to provide such a reference, especially when measurements of known molecular systems are not sufficient. However, because of the computational cost of treating core levels accurately, most calculations up to date have considered either non-periodic (cluster-type) systems or small unit cells. This has made the simulation of extended defects challenging and subject to questionable approximations, and possibly even spurious image–image interaction or finite size effects. Furthermore, the electronic structure of molecular models such as coronenes differs significantly from graphene, which can be an issue.

A prominent recent example of the value of XPS for studying graphene is in chemical functionalization, in which the pristine structure is modified by a known covalent adsorbate or a substitution. Besides substitutional doping, which we will not discuss here, the functionalization of graphene by, e.g., hydrogenation [1–2] and oxygenation [3–4] has been a topic of intense research. These treatments result in –H, –O, or –OH groups bonded to the carbon atoms, the orbital hybridization of which is changed from sp^2^ to sp^3^. This can lead to a band gap opening [3] and other interesting features [5]. To study such functional groups, along with intrinsic defects, is also vital for the spectroscopic analysis of reduced graphene oxide [6–7], which in turn is a promising avenue to the mass production of graphene.

Several intrinsic defects are relevant for graphene. Of these, the simplest are single (SV) and double vacancies (DV), along with the Stone–Thrower–Wales (STW) bond rotation. All of these have been directly observed [8] in aberration-corrected transmission electron microscopes (TEM). More extended defects (such as the 555-777 and the 5555-6-7777 double vacancy defects [9]) have also been seen, but are likely to be beam-induced. In any case, locally they do not present very different bonding environments, and thus their XPS signatures are unlikely to differ significantly from the simpler cases.

The single vacancy is different from the DV (called V_2_(5-8-5) by Banhart et al. [9]) and the STW (SW(555-777) [9]) as by necessity it presents dangling bonds. The removal of a single carbon atom from a graphene lattice leaves the three neighboring atoms with a single dangling bond each, which can be called an unreconstructed single vacancy (uSV). As this is energetically unfavorable, two of the atoms tend to form a bond between themselves and reconstruct to close a pentagon [8] in the Jahn–Teller distortion [10]. We will call this a reconstructed single vacancy, or simply SV (V_1_(5-9) [9]). However, the remaining single carbon atom still cannot satisfy its chemically reactive dangling bond, as has been directly observed by scanning tunneling microscopy (STM) in high vacuum [11].

To address these important systems, and the potential shortcomings of previous studies, we have calculated graphene core level binding energies by using density functional theory implemented with real-space grid-based projector-augmented waves in the GPAW code [12]. We applied periodic boundary conditions in large unit cells to avoid spurious image interaction effects. Furthermore, we benchmarked select results against all-electron calculations with the FHI-aims code [13] to ensure that the projector-augmented waves in GPAW described the core levels of these systems accurately.

In addition to pristine graphene, we studied *hydrogen* (-H), *dihydrogen* (2 –H), graphane-like *dihydrogen* (2 –H_opp_, i.e., two neighboring H adatoms on opposite sides of the graphene sheet [2]) *hydroxide* (–OH), *oxygen* (=O), *dioxygen* (–2O), *epoxide* (>O, [3–4]), and *carboxylic* (–COOH, [14]) functional groups. The defect structures we studied were the single vacancy (SV), double vacancy (DV) and the Stone–Thrower–Wales (STW) defects. Several modifications of the SV site were considered as well, as the dangling bond constitutes a reactive site for the absorption of molecules from the environment. As the absorption of more electronegative atoms can have a large impact on the C 1s binding energy of the neighboring carbon atom, the following adsorbates were considered: *hydrogen* (SV–H), *ketone* (SV=O), *annulene* (SV–O–, [15]), *ketone + annulene* (SV=O+–O–), *diketone* (SV=2O, possibly relevant for oxygen splitting [16]), *hydroxide* (SV–OH), and *carboxylic* (SV–COOH) groups.

We found that the projector-augmented results were in excellent agreement with all-electron calculations. In almost all cases, in which data was available, a good agreement for the C 1s levels with experimental values reported in the literature was also found [4,17–20]. As a further refinement, we considered binding energy contributions arising from up to third nearest neighbors to the functional group or defect, and plotted the resulting line shapes by using experimentally realistic broadenings. In the case of the O 1s level, the line-shape variations of graphene have not been extensively examined in experimental reports, which makes the comparison of the calculated O 1s values to literature data problematic. This is why we have focused our discussion on the C 1s energies. With this caveat, core-hole calculations with the GPAW code are a convenient and valuable tool for simulating the core level binding energies of graphene systems.

## Results

### Relaxed structures

**Figure 1:**
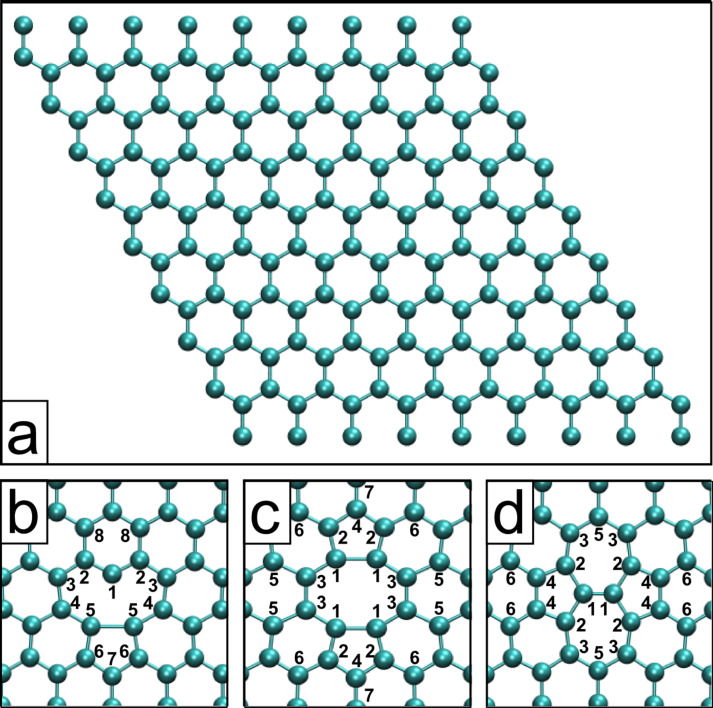
a) The 9 × 9 graphene computational unit cell. Cropped relaxed structures of the b) reconstructed single vacancy (SV), c) the double vacancy (DV), and d) the Stone–Thrower–Wales (STW) defect.

**Figure 2:**
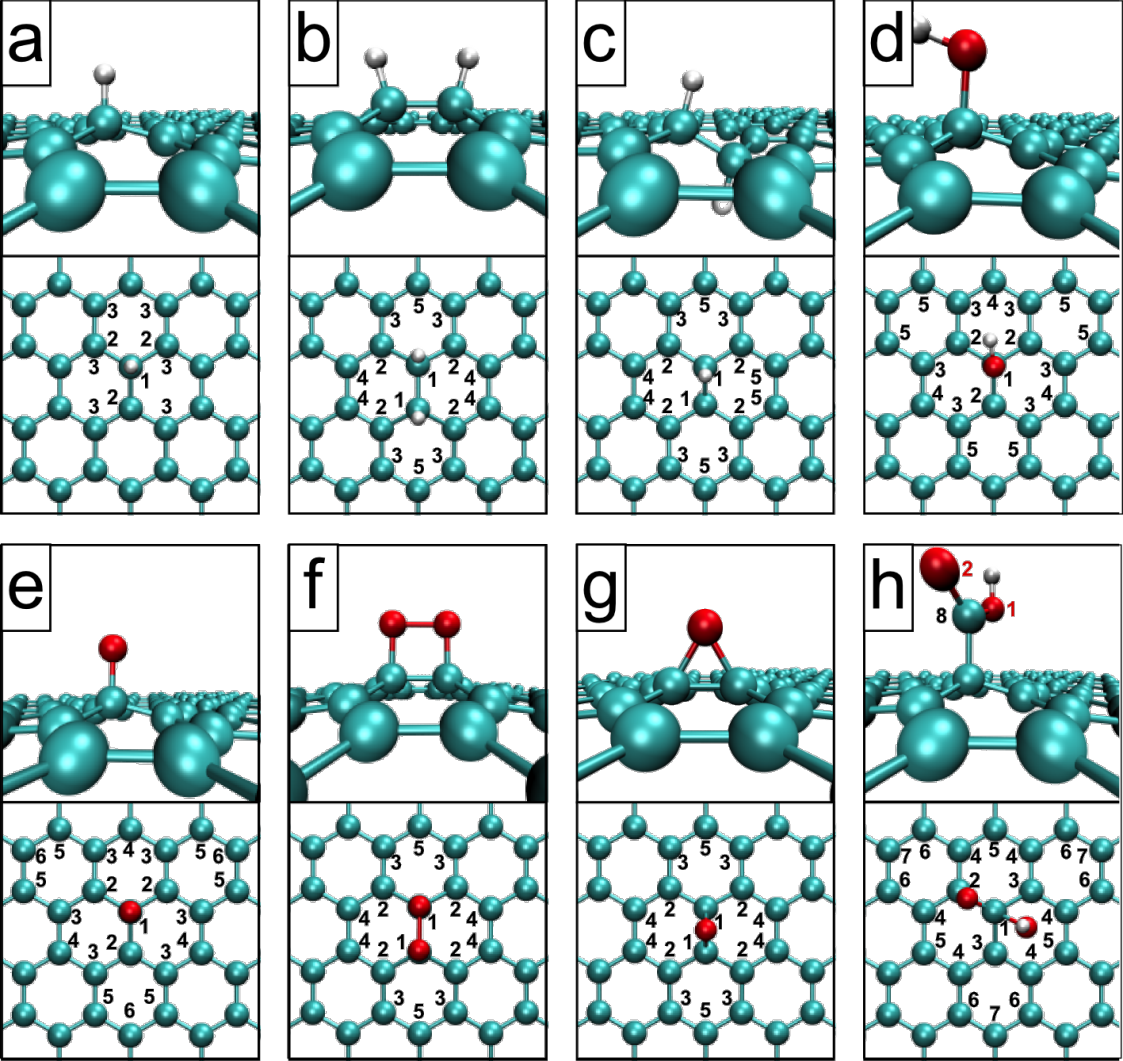
Cropped relaxed structures of functionalized graphene. The a) hydrogen (–H), b) dihydrogen (2 –H), c) graphene-like dihydrogen (2 –H_opp_), d) hydroxide (–OH), e) oxygen (=O), f) dioxygen (–2O), g) epoxide (>O), and h) carboxyl (–COOH) functionalities.

**Figure 3:**
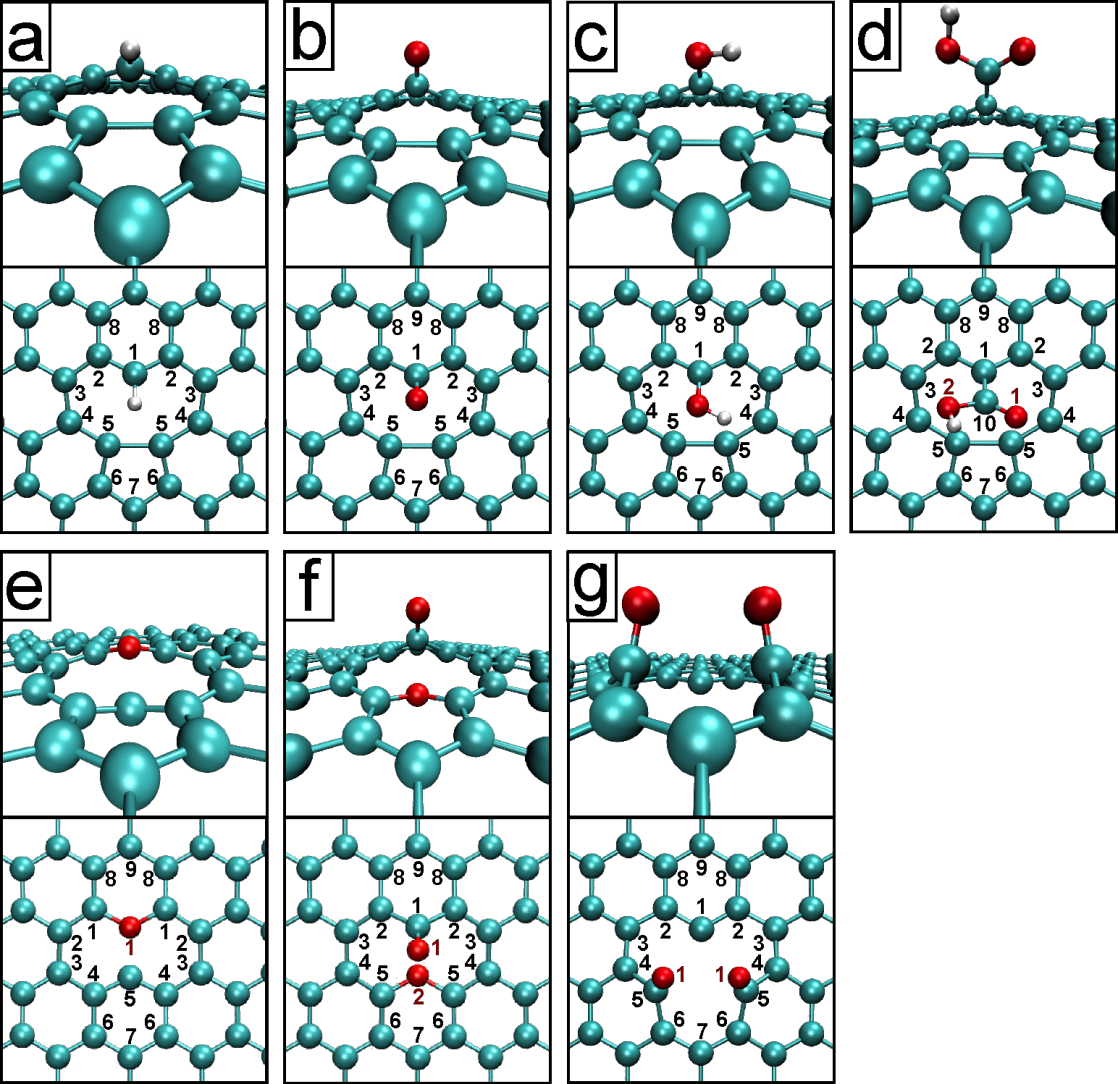
Cropped relaxed saturated single vacancy structures. The single vacancy saturated by a) hydrogen (SV–H), b) oxygen (SV–O), c) hydroxide (SV–OH), d) carboxyl (SV–COOH), e) annulene (SV–O–), f) ketone + annulene (SV=O+–O–), and g) diketone (SV=2O) groups.

The relaxed structures are shown in Figure 1, Figure 2, and Figure 3. Note that all systems were allowed to relax with no constraints, which induced a slight curvature into some of the structures to compensate for the strain induced by the local defects. The unreconstructed single vacancy spontaneously reconstructed during the geometry relaxation, by closing a pentagonal carbon ring. The bond lengths and angles of the relaxed structures match closely to what has been reported in the literature [3–4,9,14,16]. The Arabic numerals denote the target atoms of the core-hole calculations discussed below.

### Formation energies

The formation energies of the defects were calculated according to Equation 1, found in section “Computational details“ below along with the chemical potentials chosen for the missing carbon atoms and the added functional groups. The formation energy of the STW defect was calculated to be 4.99 eV, in perfect agreement with previous studies [2]. The values for the single (7.21 eV) and double vacancies (7.01 eV) are marginally lower than previously reported, which could be attributed to the unconstrained structural relaxation allowed here. Following Banhart et al. [9], it should be noted that the formation energy per atom is much lower for the double vacancy.

The formation energies of the saturated vacancy structures were calculated with respect to the bare single vacancy. The hydrogenated SV had a formation energy of −2.46 eV due to the saturation of the dangling bond. The lowest formation energies were obtained for the oxygen-saturated structures, with the ketone-saturated (SV=O) vacancy at −4.01 eV, the diketone (=2O) at −4.91 eV, the annulene-bridged vacancy (SV-O-) at –4.00 eV, and finally, the annulene plus ketone vacancy structure (SV=O + -O-), which had by far the lowest value at −8.65 eV. In agreement with a previous calculation, which used a cluster model [14], the carboxyl-saturated vacancy (SV-COOH) has a formation energy −1.62 eV compared to the SV, or 3.12 eV compared to the pristine structure.

The formation energies of the functional groups without vacancies are 1.45 eV for the -H adatom (in good agreement with a previous calculation [21]), 1.70 eV for 2 -H, 2.30 eV for the -OH, 1.20 eV for the =O, 0.74 eV for adjacent =O adatoms, and 2.07 eV for the carboxylic group -COOH. The epoxide group >O had a remarkably low formation energy of 0.3 eV, in line with the thermally reversible oxidation recently observed experimentally by Hossain and co-workers [4].

### Core level binding energies

The core level binding energies were calculated according to the delta Kohn–Sham total energy differences method [22–23] as detailed in section “Computational details”. The calculated core level binding energies for the pristine and defected graphene are shown in Table 1, for functionalized graphene in Table 2, and for the saturated vacancy configurations in Table 3. C(*) denotes a carbon atom far away from the defect (“bulk”), and “*” in the column “# of atoms” denotes that the number of such atoms depends on the defect concentration. For each configuration, we calculated the C 1s binding energies (and O 1s, where applicable) for up to third nearest neighbor C atoms from the defect to capture the significant shifts while keeping the computational effort manageable. Target atoms are denoted by Arabic numerals in Figures 1–3 with the same numeral denoting multiple equivalent atoms, and the number of atoms of each type is noted in Tables 1–3.

**Table 1:** Calculation results for the pristine and defected graphene structures. The columns show: system identifier; formation energy of the defect; target atom of the calculation (see Figure 1); number of target atoms; calculated 1s binding energy; C 1s BE shift with respect to the calculated C 1s energy of pristine graphene.

ID	*E*_form_ (eV)	atom	# of atoms	C 1s BE (eV)	BE shift (eV)

gra	0	C (*)	*	283.61	0.00

SV	7.21	C (*)	*	283.32	−0.29
		C (1)	1	281.21	−2.40
		C (2)	2	282.97	−0.64
		C (3)	2	282.87	−0.74
		C (4)	2	283.55	−0.06
		C (5)	2	283.24	−0.37
		C (6)	2	292.91	−0.70
		C (7)	1	282.51	−1.10
		C (8)	2	282.97	−0.64

DV	7.02	C (*)	*	283.39	−0.22
		C (1)	4	283.27	−0.34
		C (2)	4	282.79	−0.82
		C (3)	4	283.58	−0.03
		C (4)	2	282.43	−1.18
		C (5)	4	283.53	−0.08
		C (6)	4	283.21	−0.40
		C (7)	2	283.12	−0.49

S-W	4.99	C (*)	*	283.61	0.00
		C (1)	2	283.10	−0.51
		C (2)	4	283.16	−0.45
		C (3)	4	283.83	0.22
		C (4)	4	282.67	−0.94
		C (5)	2	283.70	0.09
		C (6)	4	283.27	−0.34

**Table 2:** Calculation results for the functionalized graphene structures. The columns show: system identifier; formation energy of the defect; target atom of the calculation (see Figure 2); number of target atoms; calculated 1s binding energy; C 1s BE shift with respect to the calculated C 1s energy of pristine graphene or the O 1s BE shift with respect to the calculated O 1s energy of epoxide/hydroxide functional groups, where applicable.

ID	*E*_form_ (eV)	atom	# of atoms	1s BE (eV)	BE shift (eV)

gra	0	C (*)	*	283.61	0.00

-H	1.45	C (*)	*	283.39	−0.22
		C (1)	1	284.10	0.49
		C (2)	3	282.78	−0.83
		C (3)	6	283.35	−0.26

2 -H	1.69	C (*)	*	283.59	−0.02
		C (1)	2	284.55	0.94
		C (2)	4	283.19	−0.42
		C (3)	4	283.58	−0.03
		C (4)	4	283.55	−0.05
		C (5)	2	283.31	−0.30

2 -H_opp_	1.30	C (*)	*	283.59	−0.02
		C (1)	2	284.36	0.75
		C (2)	4	283.16	−0.45
		C (3)	4	283.60	−0.01
		C (4)	4	283.53	−0.08
		C (5)	2	283.31	−0.30

-OH	2.30	C (*)	*	283.38	−0.23
		C (1)	1	284.81	1.20
		C (2)	3	282.35	−1.26
		C (3)	6	282.91	−0.70
		C (4)	3	282.83	−0.78
		C (5)	6	283.29	−0.02
		O	1	530.11	0.00

=O	1.20	C (*)	*	283.38	−0.23
		C (1)	1	283.93	0.32
		C (2)	3	282.35	−1.25
		C (3)	6	282.91	−0.70
		C (4)	3	282.83	−0.78
		C (5)	6	283.14	−0.47
		C (6)	3	283.16	−0.45
		O	1	526.36	−3.75

-2O	0.74	C (*)	*	283.53	−0.08
		C (1)	2	285.16	1.55
		C (2)	4	283.00	−0.61
		C (3)	4	283.44	−0.17
		C (4)	4	283.45	−0.16
		C (5)	2	282.83	−0.78
		O	2	530.29	0.18

>O	0.29	C (*)	*	283.57	−0.04
		C (1)	2	285.13	1.52
		C (2)	4	283.16	−0.45
		C (3)	4	283.56	−0.05
		C (4)	4	283.55	−0.07
		C (5)	2	283.30	−0.31
		O	1	530.11	0.00

-COOH	2.07	C (*)	*	283.34	−0.27
		C (1)	1	284.43	0.81
		C (2)	1	282.67	−0.94
		C (3)	2	282.77	−0.84
		C (4)	6	283.21	−0.40
		C (5)	3	282.89	−0.72
		C (6)	6	282.26	−0.35
		C (7)	3	283.36	−0.25
		C (8)	1	286.07	2.46
		O (1)	1	532.82	2.71
		O (2)	1	530.50	0.39

**Table 3:** Calculation results for the saturated single vacancy structures. The columns show: system identifier; formation energy of the defect; target atom of the calculation (see Figure 3); number of target atoms; calculated 1s binding energy; C 1s BE shift with respect to the calculated C 1s energy of pristine graphene or the O 1s BE shift with respect to the calculated O 1s energy of epoxide/hydroxide functional groups, where applicable.

ID	*E*_form_ (eV)	atom	# of atoms	1s BE (eV)	BE shift (eV)

SV^a^	0	—	—	—	—

SV-H	−2.46	C (*)	*	283.30	−0.31
		C (1)	1	282.11	−1.50
		C (2)	2	283.00	−0.61
		C (3)	2	282.96	−0.65
		C (4)	2	283.65	0.04
		C (5)	2	283.24	−0.37
		C (6)	2	282.78	−0.83
		C (7)	1	282.37	−0.24
		C (8)	2	283.09	−0.52

SV=O	−4.01	C (*)	*	283.47	−0.14
		C (1)	1	284.27	0.65
		C (2)	2	282.64	−0.97
		C (3)	2	283.27	−0.34
		C (4)	2	283.65	0.04
		C (5)	2	283.42	−0.19
		C (6)	2	283.01	−0.60
		C (7)	1	282.60	−1.01
		C (8)	2	283.26	−0.35
		C (9)	1	283.05	−0.56
		O	2	528.88	−1.23

SV-OH	−1.53	C (*)	*	283.35	−0.26
		C (1)	1	284.03	0.42
		C (2)	2	282.98	−0.63
		C (3)	2	283.03	−0.58
		C (4)	2	283.55	−0.06
		C (5)	2	283.28	−0.33
		C (6)	2	282.79	−0.82
		C (7)	1	282.41	−1.20
		C (8)	2	283.15	−0.46
		C (9)	1	283.13	−0.48
		O	1	531.93	1.82

SV -O-	−4.00	C (*)	*	283.20	−0.41
		C (1)	2	284.40	0.79
		C (2)	2	283.33	−0.28
		C (3)	2	282.80	−0.81
		C (4)	2	282.99	−0.62
		C (5)	1	281.25	−2.06
		C (6)	2	283.05	−0.56
		C (7)	1	283.23	−0.38
		C (8)	2	283.35	−0.26
		C (9)	1	282.92	−0.59
		O	1	532.10	1.99

SV=O + -O-	−8.65	C (*)	*	283.61	0.00
		C (1)	1	284.59	0.98
		C (2)	2	282.98	−0.63
		C (3)	2	283.62	0.01
		C (4)	2	283.75	0.14
		C (5)	2	284.89	1.19
		C (6)	2	283.80	0.19
		C (7)	1	283.54	−0.07
		C (8)	2	283.49	0.12
		C (9)	1	283.25	0.36
		O (1)	1	529.23	−0.78
		O (2)	1	532.57	2.46

SV=2O	−4.91	C (*)	*	283.28	−0.33
		C (1)	1	280.73	−2.88
		C (2)	2	282.55	−1.06
		C (3)	2	282.90	−0.71
		C (4)	2	282.39	−1.22
		C (5)	2	284.26	0.65
		C (6)	2	282.44	−1.17
		C (7)	1	283.32	−0.29
		C (8)	2	282.92	−0.69
		C (9)	1	283.03	−0.58
		O	2	528.29	−1.82

SV-COOH	−1.61	C (*)	*	283.35	−0.26
		C (1)	1	282.38	−1.23
		C (2)	2	283.01	−0.60
		C (3)	2	282.97	−0.64
		C (4)	2	283.65	0.04
		C (5)	2	283.24	−0.37
		C (6)	2	282.79	−0.82
		C (7)	1	282.36	−1.25
		C (8)	2	283.08	−0.53
		C (9)	1	283.19	−0.42
		C (10)	1	285.72	2.11
		O (1)	1	529.32	−0.79
		O (2)	1	531.83	1.72

^a^The formation energies in this table are calculated with respect to the single vacancy structure. See Table 1 for the C 1s values of this configuration.

For the all-electron FHI-aims calculations, we considered the pristine, SV, -H, and 2 -H_opp_ configurations. Although the C 1s energy of pristine graphene had a slightly different absolute value with FHI-aims (283.69 eV vs 283.61 eV), the all-electron calculations gave binding energy shifts within 10 meV of the GPAW results. This demonstrates that the use projector-augmented waves in the GPAW calculations is not a significant source of error.

### Line shapes

**Figure 4:**
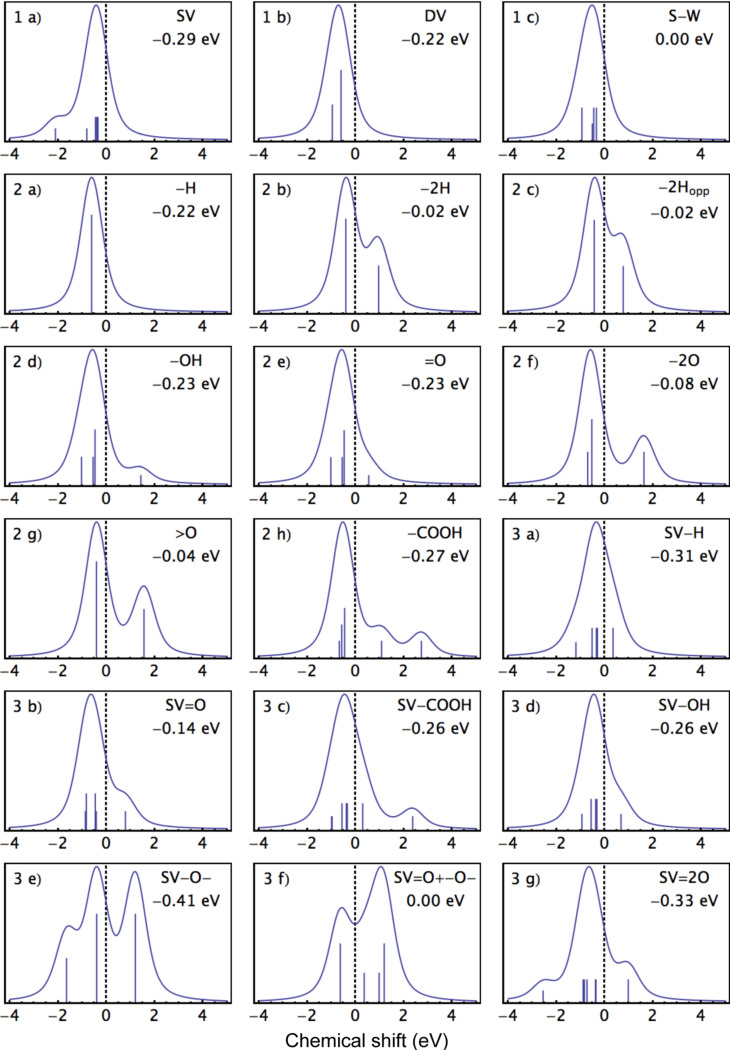
The calculated line shapes, in which peaks with shifts smaller than 0.3 eV from the system bulk value have been suppressed. The identifier in the top left corner of each panel refers to the corresponding structures in Figures 1–3, while the energy inset under the system identifier in the top right corner denotes the shift of the system bulk value compared to that of pristine graphene. The vertical lines represent the shifts of the calculated binding energies with respect to the system bulk value, weighted by the number of atoms for each calculated energy and scaled for clarity.

To help interpret the calculated core level binding energies shown in Tables 1–3, we plotted line shapes for each configuration in Figure 4. For realistic defect concentrations, most of the atoms in the system – and thus most of the photoemitted signal – will be from atoms in the “bulk” of the system. This was calculated as the C 1s energy of an atom far away from the defect, and shown as the C(*) atoms for each configuration in the tables. Since the energy resolution of most laboratory XPS spectrometers is broader than the narrow deviations that can be obtained from our calculations, we have omitted peaks with shifts less than 0.3 eV from the bulk value determined for each system from the graphical representations of the line shapes below. Thus the line shapes should be used to interpret experimental spectra only after the main C 1s peak has been subtracted. Accordingly, the plots show chemical shifts with respect to the system bulk, with weights equal to the number of atoms of each type.

Once the binding energies for the different configurations are identified, the experimental broadening must be considered. The widths of the components of these XPS peaks are defined by their Voigtian lineshape. These comprise a Gaussian broadening related to the instrumental resolution as well as vibrational effects, and Lorentzian broadening, corresponding to the lifetime of the excited electron. We have set both the Gaussian and Lorentzian amplitude to 0.3 eV in the line shapes below. This can give us a fair picture of the line shapes since this value corresponds to a good resolution and a reasonable average for the lifetimes arising from each bonding environment. In addition, we have provided the Mathematica script used to plot the line shapes as Supporting Information File 1, in which these parameters can be easily varied to match a particular experimental setup.

## Discussion

The value of the carbon 1s core level binding energy of graphite is commonly cited to be at 284.4 eV [18,24]. In the case of graphene, however, this value varies according to the substrate, on which graphene is placed or grown. Some authors have measured the C 1s of epitaxial monolayered graphene at a slightly higher value of 284.8 eV, but attribute this to a charge transfer from the SiC substrate [25]. A similar shift has been observed for the Dirac point in this system by angle-resolved photoemission spectroscopy [26]. Other authors have measured the C 1s at 284.15 eV [27] on Ir(111) and 284.2 eV on Au-intercalated Ni(111) [28], but again, charge transfer very likely contributes to the results. Since no conclusive XPS data on freestanding monolayered graphene is available so far, we have chosen to use 284.4 eV as the reference value for the graphene C 1s binding energy.

Looking at the calculated C 1s value of pristine graphene in Table 1, we can see that the computational method underestimates the binding energy by 0.8 eV. As mentioned above, the absolute values for the DFT energies will depend on the functional that was used. Errors on the order of 1 eV compared to the experimental values are typical [23]. A common practice to compare simulations to experiments is to rigidly shift the calculated values to match a well-known experimental value, which allows the prediction of core level binding energies for atomic configurations that are not known experimentally. Thus the experimentally meaningful values are the shifts of the C 1s energy with respect to the graphene bulk value, which are shown as the last column of Tables 1–3. However, it should be noted that the C 1s values for bulk atoms in certain configurations differ from the pristine graphene value by up to 0.4 eV. This shift depends on the computational unit cell size and would certainly be affected by the presence of a substrate. We have thus chosen to list the absolute shifts with respect to the pristine value in Tables 1–3, but use the shifted bulk values in the graphical representations of the line shapes shown above.

First, we must note that the C 1s energies calculated for the intrinsic defects (SV, DV and STW) are lower than the pristine graphene energy. This is only rarely seen in experiments, perhaps because of the spontaneous saturation of such sites under ambient conditions, as suggested by the negative formation energies of the saturated SV structures (Table 3). Speranza et al. [18] measured such negative shifts for irradiated graphite, and speculated that a component shifted by −0.5 eV could be due to an imbalance of electric charge around vacancies. Barinov et al. [17] explicitly calculated the dangling bond atom in the SV to have a downshift of −1.1 eV, while the two atoms in the pentagon have shifts of −0.7 eV. Our calculations give corresponding values of −2.4 and −0.37 eV. However, several other atoms surrounding the vacancy present shifts of around −0.7 eV. The binding energies for the DV and the STW defects present similar downshifts from the pristine value as in the SV case, but not quite as large.

The calculated values for the functionalized graphene systems can be found in Table 2. The C 1s value of the carbon atoms bonded to oxygen in the epoxide configuration (>O) is 1.5 eV higher than the pristine graphene value, in excellent agreement with Barinov et al., who calculated a shift of 1.6 eV [17] (although it should be noted that the experimental shift reported by Hossain et al. is slightly higher at 1.8 eV [4]). However, atoms 2 and 5 present negative shifts of −0.45 and −0.31 eV, respectively, contributing to the overall signature of these groups. For functionalities without vacancies, commonly accepted shifts in the literature [19–20] are 1.3–1.7 eV for a carbon bonded to -OH, 2.5–3.0 eV to =O, and 4.0–4.5 eV to -COOH. Looking at the values in Table 1, we find 1.2 eV for -OH, only 0.32 eV for =O, and 2.5 eV for -COOH. We note that the reference for =O actually comes from benzoquinone, which has =O functionalities at neighboring sites of the benzene ring. Thus we also considered a -2O functionality with oxygen atoms bonded to two adjacent carbon atoms. This gave a C 1s energy shift of 1.6 eV, which is closer to the literature value. However, it should be noted that the systems considered in the references above are different than those considered here, and thus one should not expect a perfect agreement. Looking at Table 2, we see that even when the C atom bonded to the functional group presents a positive shift, this is invariably compensated by negative shifts on neighboring carbons.

The calculated values for the saturated vacancy systems can be found in Table 3. For a single vacancy saturated with oxygen (SV-O), we found a shift of 0.67 eV (smaller than a reported value of 1.4 eV [17]). For the dangling bond atom in the hydroxyl group saturated vacancy (SV-OH) the C 1s is shifted by 0.42 eV, but the carbon atom at the other side of the vacancy close to the H presents a downshift of −0.29 eV, which is small but could still be experimentally observable. The carboxylic group presents large shifts of −1.23 eV for the dangling bond atom, and +2.11 eV for the carbon bonded to the two oxygens. However, considering the relatively high formation energies of the latter two structures, it should be noted that they might not represent stable ground state configurations. The diketone-saturated vacancy (SV=2O) shows a very large downshift of −2.88 eV for the dangling bond atom, and upshifts of 0.65 eV for the atoms bonded to the oxygens. The most stable ketone + annulene saturation presents large upshifts of 0.98 eV for the C bonded to the ketone O and 1.19 eV for the two C bonded to the annulene bridge O. However, atoms 2 also have moderate downshifts of −0.63 eV, complicating the peak signature. Comparing equivalent functional groups with and without the vacancy, we see that the presence of the vacancy lowers the calculated C 1s energies of the carbon atom that is attached to the functional group significantly. This is likely due to the effect of the missing electron in the p_z_ orbital of the vacancy.

Concerning the oxygen 1s core level binding energies, we chose in Table 1 to use the calculated binding energy shared by the hydroxide and epoxide functional groups as the reference with which to compare the other O 1s values. For comparing our calculations to experimental values, we will discuss the epoxide configuration, since good recent data from Hossain et al. [4] is available. In their well-characterized graphene samples functionalized with oxygen atoms convincingly in the epoxide configuration, they observed an O 1s peak at 531.9 eV. Looking at Table 1, we calculated this value to be of 530.11 eV. Thus there is a difference of 1.8 eV corresponding to a relative error of around 0.3%, the same as for C 1s. Since the absolute computational error for the O 1s energies is different than for the C 1s energies we cannot use the same shift for the two, and we have much less information about the correct O 1s values in our particular case. However, the relative shifts between our calculated O 1s values are expected to be accurate and useful if an experimental baseline can be established in a specific study.

## Conclusion

We have calculated the core level binding energies of both pristine and defective monolayered graphene functionalized with oxygen- and hydrogen-based adsorbates in a large and periodic unit cell. We have shown that the use of the projector-augmented wave method does not introduce significant errors in the treatment of the core electrons compared to all-electron calculations. The computationally efficient and scalable GPAW code is thus well suited for calculating core level binding energy shifts for graphene-based systems. However, higher levels of theory or more advanced functionals could certainly improve the absolute energy values. Because good agreement was obtained with experimental data found in the literature as far as it was available, we envisage that the calculations presented here will be especially useful for predicting the X-ray photoelectron spectroscopy signatures for novel structures for which such data is not available.

## Computational details

Density functional theory was used as implemented in the GPAW simulation package [12]. The projector-augmented wave method [29] was used with frozen core electrons, and exchange and correlation was estimated by the Perdew–Burke–Ernzerhof (PBE) generalized gradient approximation [30]. Periodic boundary conditions were applied with a Monkhorst–Pack [31] *k*-point mesh up to 5 × 5 × 1 *k*-points.

### Convergence checks

First, the pristine graphene lattice distance *a*_0_ and the GPAW grid spacing parameter *h* were carefully converged with respect to the total energy. The converged parameters were *a*_0_ = 2.443 Å and *h* = 0.19 Å. Next, the carbon 1s core level binding energy (using total energy differences) of a carbon atom in graphene was converged with respect to the unit cell size. The use of a sufficiently large unit cell is important to avoid spurious interactions with periodic images of the core hole. The maximum unit cell size for which the core-hole calculation could be completed with the available computational resources was 11 × 11. However, the C 1s energy was fully converged already for a 9 × 9 unit cell (a total of 162 atoms) when employing 3 × 3 × 1 *k*-points in the calculation. This convergence was checked to be valid also for more extended defects. A vacuum distance of 8 Å in the direction perpendicular to the graphene plane was sufficient to ensure convergence in all cases, including the highly non-planar -COOH functional group. All structures were allowed to fully relax so that the maximum forces reached less than 0.01 eV/atom. The all-electron projected density of states of the pristine graphene system reproduced all of the expected features of graphene, including the Dirac cones and the semi-metallic nature of a graphene monolayer. Similarly for FHI-aims, the convergence of both the total energies and the studied core level binding energies with respect to the computational parameters was ensured.

### Formation energies

The formation energies *E*_form_ of the various configurations were calculated as

[1]
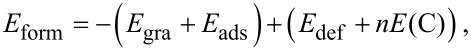


where *E*_gra_ is the total energy of pristine graphene (for functional groups and vacancies) or the total energy of graphene with a single vacancy (for saturated single vacancies), *E*_def_ is the total energy of the system with a defect, *E*(C) is the energy for each of the *n* removed carbon atoms (equal to *E*_gra_/*N*, where *N* is the number of atoms; in this case 162), and *E*_ads_ is the energy of the adsorbants.

The energies of missing carbon atoms were calculated as the energy of the pristine graphene sheet divided by the number of atoms, 1492.312 eV / 162 = 9.212 eV. The energies of added hydrogen and oxygen atoms were determined with respect to the chemical potentials of H_2_ and O_2_ molecules, which we calculated at 6.755 eV / 2 = 3.377 eV for hydrogen, and 9.137 eV / 2 = 4.569 eV for oxygen. For the COOH functionalities, we calculated the energy of a HCOOH molecule in vacuum by using the same unit cell and parameters as in the graphene calculations [14], which yielded an energy of 30.213 eV, and subtracted half the H_2_ molecule energy. In order to calculate the formation energies of the OH functionalities, we used *E*_OH_ = *E*_H2O_ − *E*_H2_/2 [32], with the energy of the H_2_O molecule calculated to be 14.336 eV.

### GPAW core-hole calculation

The total energy of a system before photoemission is a sum of the energy of the X-ray photon, *h*ν, and the energy of the target system in its initial state, *E*_i_. After the photoemission, the total energy is equal to the kinetic energy of the emitted photelectron, *E*_k_, plus that of the ionized system in its final state, *E*_f_. We thus have *h*ν + *E*_i_ = *E*_k_ + *E*_f_. The binding energy, *E*_b_, of the 1s electron is given by the difference between the energies of the X-ray photon and the emitted photelectron: *E*_b_*_=_ h*ν − *E*_k_, which leads to *E*_b_ = *E*_f_ − *E*_i_, the difference between final and initial energies of the target system.

For the DFT calculations, we used the real-space grid-based projector-augmented wave (GPAW) code [12]. Recently, core-hole calculations that utilize a delta Kohn–Sham (∆K–S) total energy differences method were implemented into GPAW by Ljungberg et al. [22–23], and into SIESTA by García-Gil et al. [33]. The core-hole setup (similar to a pseudo-potential) is created by using a spin-paired atomic calculation with the occupation of the core orbital decreased by one and held fixed. This setup is used to replace the target atom in a system of interest in the calculation. To obtain correct exchange–correlation effects, the 1s core spin densities are scaled to make the hole confined to spin up, which is an approximation that works very well for the case of small atomic number elements such as carbon and oxygen with only one core state, but requiring a spin-polarized calculation for the system of interest. A similar methodology, however employing pseudo-potentials [17], was previously used to study oxidized graphene.

The energy of the core level excitation was determined in the ∆K–S procedure, in which the total energy difference between the ground state and the first core ionized state is calculated. The core electron is removed from the 1s state and introduced into the valence band to ensure the neutrality of the unit cell. For metallic systems this is a very reasonable approach since the screening of the core hole is very efficient and the extra electron would be introduced at the Fermi level; however, for systems with large band gaps, this procedure could lead to large errors. Although the energy will depend on the exchange–correlation functional being used, the method should give consistent results for all atoms of the same kind. Since the C 1s level of graphite is well known experimentally, a rigid shift of the calculated energy scale to match it for the pristine defect-free system is applied to all C 1s energies calculated, which allows for a comparison of the results to experimental measurements. For O 1s, no unambiguous reference energy is present in all samples that could be used to shift the calculated O 1*s* energies.

### FHI-aims all-electron calculations

Finally, to confirm that the use of the projectors did not introduce errors in the treatment of the core level energies, we performed additional calculations for selected systems using the all-electron code FHI-aims [13], also with the PBE functional, and compared the C 1s energy values to the corresponding GPAW calculation. The core level energies were calculated by comparing the relaxed total energies of a system with or without a core hole – described by an explicitly empty 1*s* core orbital in the case of FHI-aims – on an atom of interest.

## Supporting Information

File 1 Mathematica script used for plotting the line shapes shown in Figure 4.
